# Genetic profiles of plasmacytoid (BDCA-4 expressing) DC subtypes-clues to DC subtype function *in vivo*

**DOI:** 10.1186/2162-3619-2-8

**Published:** 2013-03-09

**Authors:** Stephen H Wrzesinski, Jan L Fisher, Marc S Ernstoff

**Affiliations:** 1 Department of Internal Medicine Dartmouth-Hitchcock Medical Center, One Medical Center Drive, Lebanon, NH, 03756, USA; 2 Medical Oncology Immunotherapy Program, Section of Hematology and Oncology, Dartmouth-Hitchcock Medical Center, Norris Cotton Cancer Center, One Medical Center Drive, Lebanon, NH, 03756, USA; 3 St. Peter’s Cancer Care Center, 317 S. Manning Blvd, Suite 220, Albany, NY, 12208, USA

**Keywords:** Plasmacytoid dendritic cells, Gene expression, Granzyme B

## Abstract

Among the dendritic cell (DC) subsets, plasmacytoid DC’s (pDC) are thought to be important in the generation of both antiviral and antitumor responses. While pDC may be useful in developing dendritic cell-based tumor vaccines, the low frequency of these cells in the peripheral blood has hampered attempts to understand their biology. To provide better insight into the biology of pDC, we isolated these unperturbed cells from the peripheral blood of healthy donors in order to further characterize their gene expression. Using gene array technology we compared the genetic profiles of these cells to those of CD14+ monocytes isolated from the same donors and found several immune related genes upregulated in this cell population. This is the first description, to our knowledge, of gene expression in this subset of DCs obtained from the peripheral blood of adult human donors without exposure *in vitro* to cytokine or growth factors. Understanding the natural genetic profiles of this dendritic cell subtype as well as others such as the BDCA-1 expressing myeloid DCs may enable us to manipulate these cells *ex-vivo* to generate enhanced DC-based tumor vaccines inducing more robust antitumor responses.

## Introduction

Dendritic cells are paramount in generating cell-mediated and humoral immune responses and have been utilized for cancer vaccines against melanoma, prostate cancer, renal cell carcinoma, and non-Hodgkin’s lymphoma with varying results, including an FDA approved therapy for prostate cancer patients [1-5]. The DC’s used in many of these trials are generated from a heterogenous group of immature peripheral blood DC’s and CD14^+^ cells obtained from cancer patients. These cells are expanded *ex vivo* in the presence of GM-CSF and Interleukin-4, subjected to antigen loading, maturation and subsequently reintroduced into the donor. The heterogeneity of the DCs confounds the overall results observed in the patients’ antitumor responses in these trials.

Although there can be plasticity of function between DC subtypes [6], in general, myeloid DCs (mDC) and plasmacytoid DCs (pDC) are considered to induce cell-mediated (TH1 type) and humoral (TH2) responses respectively. Blood DC antigen (BDCA) 1 and 4 are preferentially expressed on mDC and pDC respectively and can be exploited to isolate pure populations of these cell types [7]. While mDC have been characterized both in healthy donors and cancer patients [8], the low frequency of pDC in the peripheral blood compartment has prevented extensive evaluations of this cell population. Little is known about the *de novo* state of pDC. Until recently [9], peripheral blood pDC have not been well-characterized and the biology of these cells has been established using either murine or human cells expanded in culture from CD34+ precursors [10]. Because of their ability to produce high levels of type I (α and β) interferons, pDC are thought to play a role in augmenting antiviral responses [11]. More recently these cells have also been demonstrated to synergize with mDCs to induce antigen-specific antitumor responses against ova-expressing murine thymomas [12] and basal cell cancers [13]. These studies suggest that initiating DC expansion from pure DC precursors (pDCs or mDCs) would be an attractive strategy for human DC vaccine development. Understanding the biology of *de novo* DC subsets could help to determine the optimal use of these cells in tumor vaccine studies. Therefore, we isolated and evaluated the gene expression profiles of human pDCs.

## Materials and methods

### pDC isolation and purification by MACS and FACS

After signing consent per Protocol D9726 (Committee for the Protection of Human Subjects#12756), healthy adult donors were tested for viral serologies (HIV, Hepatitis) and underwent leukapheresis using a Cobe Spectra Apheresis System (Lakewood, CO) if viral titers were negative. Each leukopak was then subjected to Ficol-gradient isolation and resulting peripheral blood mononuclear cells (PBMC) were stored in RPMI containing autologous serum overnight at 4°C.

The following day 1.1 x 10^9^ donor PBMC were stained with defined iron-conjugated antibodies and magnetically sorted as per Miltenyi Biotec protocol on the autoMACS. (Miltneyi Biotec, Auburn CA). The labeled cells were subjected to FACS using a FACStar Plus cytometer by pre-gating on the subset of CD3- and CD20- cells, and then gating on the BDCA-4 positive cells in this subset. Cell populations with at least 95% purity by FACS analysis were used for subsequent experiments.

### Gene array evaluation of BDCA-4 cells and confirmation of upregulated genes by RT-PCR

RNA was extracted using the Quiagen RNeasy method (Quiagen, Valencia, CA). The RNA was subsequently labeled and hybridized to the HU133A gene chip using the method established by Affymetrix and run on the Affymetrix system (Affymetrix, Santa Clara, CA). The gene expression data was subjected to analysis by the Iobion Gene Traffic ® software as described below.

Changes in expression levels of genes of interest were confirmed by RT-PCR using RNA extracted from two of the isolated BDCA-4+ DC cell samples and the autologous CD14+ cell samples used for the gene array comparisons. In brief, RNA was isolated from FACS sorted BDCA-4 and CD14 positive cells using standard Qiagen-RNeasy protocol. RNA was eluted with 30 μl of RNase-free water and stored at −80°C. The cDNA was synthesized using iscript cDNA kit (Bio-Rad, Hercules, CA). The Medline database was used to obtain mRNA gene sequences. Primers for all of the upregulated genes confirmed by RT-PCR (ie, Granzyme B, Chemokine like receptor 1, TLR-7) were designed using Primer3 Output, an on-line primer-design program. Primer sequences were as follows:

TLR7 forward AAT GTC ACA GCC GTC CCT AC

TLR7 reverse TTA TTT TTA CAC GGC GCA CA

GZMB forward ATG CAA CCA ATC CTG CTT CT

GZMB reverse TTA TGG AGC TTC CCC AAC AG

Cmklr1 forward CGT CTT CCT CCC AAT CCA TA

Cmklr1 reverse AAG AAA GCC AGG ACC CAG AT

My iQ Single Color Real-Time PCR Detection system was used to perform real-time RT-PCR (Bio-Rad, Hercules, CA). Amplification of β-actin was used as an endogenous reference gene.

### Data analysis

CellQuest software was used to analyze the purity of the BDCA-4 cells obtained by MACS and FACS.

Differential gene expression was evaluated by Iobion Gene Traffic ® software by using differential gene expression of greater than or less then 3 fold between groups [14] as described below. CD14 monocytes obtained from the same donor served as the baseline gene array profile for all analyses (see Gene Expression Omnibus [GEO] GSE11943 for full sets of gene array data).

Affymetrix image and data files were directly imported in GeneTraffic Uno Microarray Data Management and Analysis Software Version 3.2, Iobion Informatics LLC, La Jolla, CA. Data was normalized by transforming with Robust Multichip Analysis (RMA) [15]. Fold change values were generated by comparing BDCA-4 chips to CD14 chips from the same donor. The dataset was filtered to remove genes without at least one instance of expression change that was greater than or less than an absolute fold change value of 3 across 4 separate gene arrays (one array per four separate BDCA-4 cell isolations). The resulting genes were analyzed using Significance Analysis of Microarrays (SAM) [16] to determine reproducibility across replicates as well as identify significant expression changes between cell types. Using a two-class paired test with a significance cutoff of 0.001%, 1232 genes were identified that were significantly up or down-regulated when compared to CD14 cells. Subsets of differentially expressed genes were further analyzed using Onto-Express [17] to identify significantly represented ontology groups.

## Results/discussion

**Figure 1:**
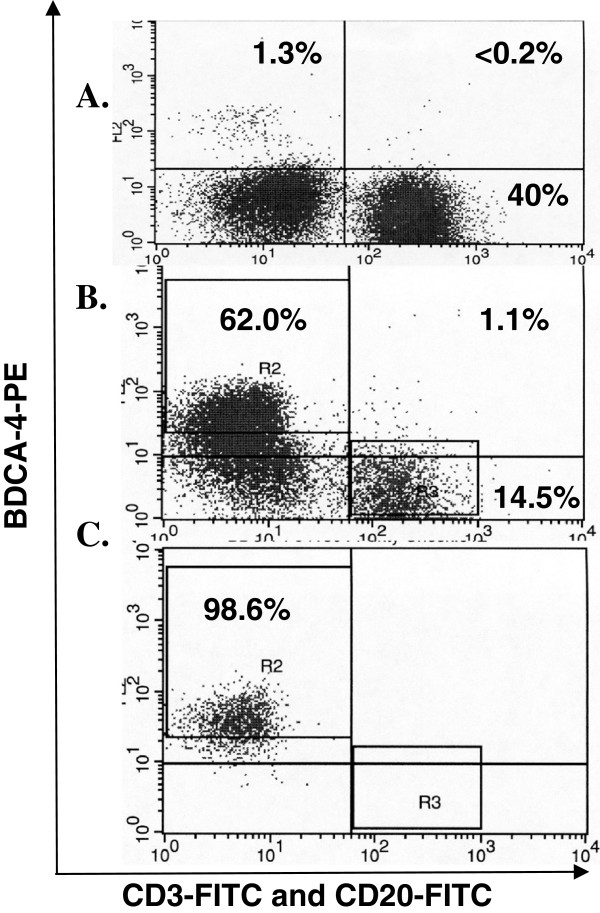
**Separation of BDCA-4 subpopulation by MACS and FACS.** Representative Flow Cytometry plot evaluating the purity of BDCA-4 populations from Ficol-separated PBMCs obtained from normal healthy donors. Leukocytes stained with BDCA-4-PE and the lymphocyte markers CD3-FITC and CD20-FITC and evaluated by flow cytometry: Prior to sorting (**A**), after Magnetic Bead Sorting (MACS) (**B**), and after MACS followed by Fluorescence-Activated Cell Sorting (FACS) (**C**). R2 indicates the left upper quadrant sorting gate, R3 indicates gate for lymphocytes removed by FACS.

We utilized the cell-specific surface expression of BDCA-4 antigens on pDCs to reproducibly obtain these populations at greater than 95% purity using MACS followed by FACS (Figure 1C). FACS sorting for BDCA-4+ cells not co-expressing the B cell marker CD20 or the T cell marker, CD3, eliminated contaminating lymphocytes retained following the first isolation (Figures 1A and 1B).

Gene arrays to evaluate the gene expression in DC’s during maturation *in vitro* have revealed upregulation of genes involved in cell adhesion and motility, immune response, growth control and lipid metabolism affecting cell growth and differentiation, signal transduction, ion channel activities and membrane function [18-20]. More recently Lindstedt et al. published the gene family clustering of human blood and tonsillar DC subsets from healthy children undergoing tonsillectomy using Affymetrix gene arrays [21]. We chose the Affymetrix system to assess gene profiles of pure populations of unstimulated *de novo* BDCA-4 cells obtained from consented healthy adult donors.

**Figure 2:**
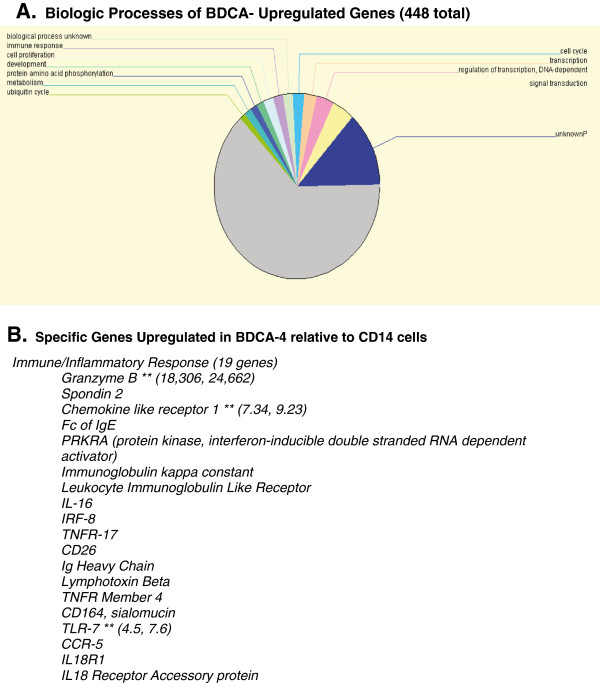
**Summary of BDCA-4 dendritic cell genes upregulated (A) and genes involved in the immune response.** (**A**) Biologic processes of genes upregulated at least three fold using the OntoExpress software as discussed in the materials and methods section. (**B**) List of specific genes upregulated in BDCA-4 cells relative to CD14+ cells. ** Genes confirmed to be upregulated using RT-PCR as discussed in the materials and methods section. Numbers in parentheses indicate two separate values of fold upregulation of the selected upregulated genes confirmed by RT-PCR.

While gene arrays of DC’s have been compared to several immune cells, including T-lymphocytes, B-lymphocytes and macrophages [18], we compared the genetic profiles of four separate isolations of BDCA-4^+^ cells from healthy donors to CD14^+^ cells devoid of BDCA-4 cells as this cell population is commonly used as DC precursors in cancer vaccine trials. Out of over 22,000 genes assessed between both populations of cells, 448 genes including the BDCA-4 gene were upregulated at least three-fold compared to those expressed in CD14+ cells (see GSE11943 for gene array datasets). While the majority of the genes encoded proteins of unknown function, the remaining upregulated genes involved several biologic processes including the immune response, cell proliferation, development, protein amino acid phosphorylation, metabolism, the ubquitin cycle, cell cycle, transcription and signal transduction pathways (Figure 2A). Nineteen of these genes were involved in the immune and inflammatory response (Figure 2B). Interestingly, the Granzyme B gene was overexpressed over 200-fold in all four BDCA-4 cell samples with RT PCR confirming an 18,000 to 25,000 fold upregulated expression of this gene relative to CD14 cells (Figure 2B). Granzyme-B is expressed by innate and adaptive immune effector cells and plays key roles in the destruction of tumor cells and cells infected with intracellular pathogens such as viruses [22]. Rissoan et al. identified high levels of mRNA expression in both resting and activated pDC obtained from cord blood and tonsillar tissue [23] and this enzyme is upregulated by a pDC leukemia [24]. Our results reinforce the recently published data reported by Tel J et al. which indicates that this DC cell subtype can be skewed as a killer DC subset when exposed to viral vaccines [25]. The gene array data demonstrating overexpression of Granzyme B at baseline suggests a genetic potential for effector function by pDC *in vivo*.

Our data demonstrate unique immune-related genes expressed in adult peripheral blood plasmacytoid dendritic cell subtypes relative to autologous CD14 monocytes and complement the previously reported differential gene expression of DC subsets obtained from the peripheral blood and tonsils of children [21].

Interestingly, we found a slightly different pDC gene expression profile compared to that reported by Lindstedt et al. While our results also demonstrate several immune-related genes upregulated in this subset including IL18R1, IL3RA (CD123) and TLR-7, additional upregulated genes not reported by Lindstedt et al. included the leukocyte-associated immunoglobulin like receptor, IL-18 receptor accessory protein, and lymphotoxin beta. One limitation of our study was that with the human HU133A chip we could not assess the previously demonstrated upregulated genes CXCR3, CLECSF7 and TLR-9 genes [21].

There are a number of reasons to explain why our results differ from those previously published. First, we used the more stringent cutoff of greater than or equal to three-fold up- or down-regulation in our analyses to further eliminate potential gene signal noise while Lindstedt et al. used ≥ two-fold [21]. Also, the pDC used in Lindstedt’s study were obtained from children who underwent tonsillectomies [21] while our pDC came from the peripheral blood of healthy adults. Aging can affect both the phenotype and the function of human monocytes and leukocytes [26]. Additionally, cord blood-derived monocytes and adult monocytes have been demonstrated to have differential gene profiles at rest and when stimulated with LPS [27]. Therefore it is conceivable that the unstimulated pDC from our older adult donors would express different levels of immune-related genes when compared to the same subtypes obtained from children undergoing tonsillectomies.

In summary, we have utilized FACS and MACS to successfully isolate highly purified populations of pDC from healthy donors and present the genetic signatures of these cells. We have identified several upregulated genes in pDC when compared to the CD14^+^ monocytes with the gene encoding the effector enzyme, Granzyme B, being the most upregulated in our isolated pDC, suggesting an effector function of these cells in the peripheral blood of healthy adults. The genetic profiles from the pDC in our studies differed from those published by Lindstedt et al. This may be due to a combination of setting different cutoff points for considering significant up- and down-regulation of gene expression as well as using cells obtained from older donors. Our results yield further information regarding unstimulated pDCs *de novo* providing an additional foundation for developing human DC tumor vaccines.

## Competing interests

The authors declare no competing financial interests.

## Authors’ contributions

SW and ME designed the research. SW, and JF performed the research. SW analyzed the data. SW and ME wrote the paper. All authors checked and approved the final version of the manuscript.
